# Challenges of Sexuality Expression in Individuals with Autism Spectrum Disorder

**DOI:** 10.1192/j.eurpsy.2024.152

**Published:** 2024-08-27

**Authors:** M. M. Figueiredo, P. Afonso, V. Vila Nova

**Affiliations:** ^1^Psiquiatria; ^2^Centro Hospitalar Setúbal, Setúbal, Portugal

## Abstract

**Introduction:**

Sexuality, although an essential component of human health, remains a controversial topic shrouded in stigma, particularly in the context of neurodiversity, which includes autism spectrum disorder (ASD), where the expression of sexuality presents unique challenges. Autism and sexuality is a complex and multifaceted topic that involves understanding the unique ways in which individuals on the autism spectrum experience and express their sexuality.

**Objectives:**

The purpose of this work is to address the complexity of the biopsychosocial sexuality components of people with autism, promoting a shift in the medical perspective, societal attitudes, and supporting greater inclusion of these individuals in current discussions regarding this area of human behavior and experience.

**Methods:**

Evidence-based review, through research conducted on PubMed and selection of the most relevant studies on this topic, published in the last decade.

**Results:**

Sexuality in autism is now recognized as a normative and integral aspect of development and functioning. Existing research suggests that most individuals with ASD display a clear interest in sexuality and relationships, with a study reviling that 96% of the ASD sample expressed an interest in sexuality. Individuals with high autistic traits tended to identify themselves more times as bisexual or presented a sexuality not definable within the categories of heterosexual. The relationship between autism and gender dysphoria is an area of ongoing research and discussion. Studies have suggested a higher prevalence of gender diverse identities and experiences within the autism community compared to the general population. Various hypotheses have been proposed to explain the increased gender and sexual diversity among individuals with autism. People with ASD may face unique challenges when it comes to their sexuality. The impairments in social skills and communication central to ASD potentially impact an autistic individual’s expression and experience of sexuality by affecting their abilities to understand and interpret social cues, emotions, and nonverbal behaviors of others. Importantly, such individuals may be more vulnerable, as they may have different or even limited understanding of boundaries and consent. To address these challenges, it is important to acknowledge and respect the diversity of sexual experiences and desires among individuals with neuro(bio)logical differences. This can be done by providing accurate and inclusive sex education, creating safe spaces for such individuals to explore and express their sexuality, and working to address discrimination and abuse in intimate contexts.

**Conclusions:**

Recognizing and respecting this diversity and fostering inclusive and accepting environments, we can help individuals with neurological differences to fully express and explore their sexuality and have satisfying sexual lives.

**Disclosure of Interest:**

None Declared

